# Efficacy and Safety of Intravitreal Therapy in Macular Edema Due to Branch and Central Retinal Vein Occlusion: a Systematic Review

**DOI:** 10.1371/journal.pone.0078538

**Published:** 2013-10-25

**Authors:** Amelie Pielen, Nicolas Feltgen, Christin Isserstedt, Josep Callizo, Bernd Junker, Christine Schmucker

**Affiliations:** 1 University Eye Hospital, Albert Ludwig University, Freiburg, Germany; 2 University Eye Hospital, Medical School of Hannover, Hannover, Germany; 3 University Eye Hospital, Georg-August-University, Goettingen, Germany; 4 German Cochrane Centre, Institute of Medical Biometry and Medical Informatics, Department of Medical Biometry and Statistics, University Medical Centre, Freiburg, Freiburg, Germany; Massachusetts Eye & Ear Infirmary, Harvard Medical School, United States of America

## Abstract

**Background:**

Intravitreal agents have replaced observation in macular edema in central (CRVO) and grid laser photocoagulation in branch retinal vein occlusion (BRVO). We conducted a systematic review to evaluate efficacy and safety outcomes of intravitreal therapies for macular edema in CRVO and BRVO.

**Methods:**

**And Findings**: MEDLINE, Embase, and the Cochrane Library were systematically searched for RCTs with no limitations of language and year of publication. 11 RCTs investigating anti-VEGF agents (ranibizumab, bevacizumab, aflibercept) and steroids (triamcinolone, dexamethasone implant) with a minimum follow-up of 1 year were evaluated.

**Efficacy: CRVO:**

Greatest gain in visual acuity after 12 months was observed both under aflibercept 2 mg: +16.2 letters (8.5 injections), and under bevacizumab 1.25 mg: +16.1 letters (8 injections). Ranibizumab 0.5 mg improved vision by +13.9 letters (8.8 injections). Triamcinolone 1 mg and 4 mg stabilized visual acuity at a lower injection frequency (-1.2 letters, 2 injections).

**BRVO:**

Ranibizumab 0.5 mg resulted in a visual acuity gain of +18.3 letters (8.4 injections). The effect of dexamethasone implant was transient after 1.9 implants in both indications.

**Safety:**

Serious ocular adverse events were rare, e.g., endophthalmitis occurred in 0.0-0.9%. Major differences were found in an indirect comparison between steroids and anti-VEGF agents for cataract progression (19.8-35.0% vs. 0.9-7.0%) and in required treatment of increased intraocular pressure (7.0-41.0% vs. none). No major differences were identified in systemic adverse events.

**Conclusions:**

Anti-VEGF agents result in a promising gain of visual acuity, but require a high injection frequency. Dexamethasone implant might be an alternative, but comparison is impaired as the effect is temporary and it has not yet been tested in PRN regimen. The ocular risk profile seems to be favorable for anti-VEGF agents in comparison to steroids. Because comparative data from head-to-head trials are missing currently, clinicians and patients should carefully weigh the benefit-harm ratio.

## Introduction

Macular edema is the main cause of visual impairment in central retinal vein occlusion (CRVO) and branch retinal vein occlusion (BRVO) [1,2]. In recent years, anti-inflammatory and anti-angiogenic therapeutic strategies have been used to target vascular permeability and leakage to reduce macular edema and improve vision. 

Among corticosteroids, triamcinolone acetonide, dexamethasone and fluocinolone have shown potential to reduce edema in RVO [3–6]. However, their drawbacks are known side effects, such as cataract progression and rise of intraocular pressure (IOP). The off-label triamcinolone acetonide is crystalline and is commercially available as Kenalog (Kenalog, 40 mg/mL, Bristol-Myers Squibb, Princeton, NJ), Trivaris (Pharm Allergan Inc., Irvine California) or prepared for injection with methocel [7]. Dexamethasone is more potent and soluble in comparison to triamcinolone and various attempts have been made to construct a slow-release device. Ozurdex™ (Pharm Allergan Inc., Irvine California) was the first dexamethasone implant that was approved by the Food and Drug Administration (FDA) for intraocular use in chronic uveitis and macular edema due to RVO in June 2009. Ozurdex shows an anti-edematous effect up to 6 months [8,9]. A fluocinolone implant is currently under investigation that is supposed to even last for up to 3 years [6].

Beside corticosteroids, progress in anti-angiogenic drug development provides us with multiple new therapeutic agents based on a concept of modified antibodies versus vascular endothelial growth factor (VEGF) and related molecules. For example, pegaptanib sodium, a 40-kDa RNA aptamer, binds to the isoform 165 of VEGF and was first approved for intravitreal therapy in wet age-related macular degeneration (AMD) [10,11]. A possible effect on visual acuity in macular edema due to CRVO and BRVO was investigated but pegaptanib did not receive approval for these indications [12,13]. Currently, the most frequently used anti-VEGF agents are ranibizumab (Lucentis™, Genentech, Inc., South San Francisco, CA, and Novartis Pharma AG, Basel, Switzerland) and bevacizumab (Avastin™, Genentech, Inc., South San Francisco, CA, and Roche, Basel, Switzerland). Ranibizumab is an antibody fragment with a high binding affinity towards all forms of VEGF. It has been approved for intravitreal therapy of wet AMD, diabetic macular edema and RVO by the FDA and European Medicines Agency (EMA) between 2006 and 2012 (depending on the indication). Bevacizumab is widely used as off-label intravitreal therapy. The cost difference to ranibizumab is striking. Large Head-to-head studies sponsored by the American and British public authorities have been conducted to compare both drugs in wet AMD (CATT [14] and IVAN [15], respectively). One and two year data of these trials showed no inferiority. Despite the approval of ranibizumab for macular edema in RVO, we assume that off-label bevacizumab is used as frequently in RVO as in AMD. This assumption is supported by a vast number of publications on the use of bevacizumab for RVO in clinical settings (e.g. [16–18]). The latest developed anti-angiogenic drug is aflibercept (VEGF-trap eye, Eylea™, Regeneron Pharmaceuticals, Inc., and Bayer Pharma AG, Berlin, Germany), a 115-kDa decoy receptor fusion protein, composed of the second domain of human VEGF receptor 1 and the third domain of VEGF receptor 2, which are fused to the Fc domain of human IgG1 [19,20]. A recent publication reported that the binding affinity for this drug is even higher than for ranibizumab and bevacizumab, respectively [21]. Aflibercept was first approved for intravitreal therapy of wet AMD in 2011. FDA approval for RVO was granted in September 2012. EMA approval is expected for 2013. 

Most of this pharmaceutical research was conducted at the same time. Notably, the multicenter randomized controlled trials (RCTs) leading to FDA approval in RVO were conducted in parallel, which resulted in homogenous control groups using observation and/or sham injection in CRVO and sham injection and/or grid laser photocoagulation in BRVO [9,20,22,23]. These research proceedings, however, resulted in a lack of head-to-head studies, comparing the various drugs to each other. Currently the ophthalmologist can choose between different therapeutic options to treat vision loss due to macular edema in RVO. Some of the therapeutic options are approved, but others are used off-label. To support the ophthalmologist’s decision between the different therapeutic options and to calculate the accompanying risks, we conducted a systematic review to investigate and compare the efficacy and safety of all therapeutic agents currently used in intravitreal therapy in RVO with a follow-up of at least 12 months. 

## Methods

### Search Strategy

We searched Medline, Premedline, Embase and the Cochrane library from inception until November 2012. The search strategy was based on combinations of medical subject headings and keywords and was not restricted to specific languages or years of publications. The search strategy used in Medline is presented in Text S1. Search strategies for other databases were modified to meet the requirements of each database. The searches were supplemented by hand searching the bibliographies of included studies. Currently conducted RCT’s on intravitreal treatment of macular edema in RVO were searched in the register for clinical trials (http://clinicaltrials.gov) and in the WHO International Clinical Trial Registry Platform (http://www.who.int/ictrp/en/).

### Inclusion and exclusion criteria

We included RCTs which investigated intravitreal ranibizumab, bevacizumab, pegaptanib, aflibercept, triamcinolone, dexamethasone, or fluocinolone as monotherapy in comparison to any non-surgical control group (observation or sham injection in CRVO, sham injection and/or grid laser photocoagulation in BRVO). Trials which compared one substance to another or to grid laser photocoagulation were also included. To address long-term efficacy and systematic adverse effects, one year follow-up data (or longer) had to be available.

Studies were excluded if participants received a combination of intravitreal pharmacological agents within the same eye (e.g. ranibizumab and triamcinolone), if both eyes of the same person were treated as study eyes, and if less than 10 patients per arm were enrolled. Studies which treated patients who had already been treated within 3 months using one of the substances were excluded. To account for the 2 different indications, study results had to be attributable to either BRVO or CRVO separately. 

### Data Extraction and Quality Assessment

Titles and abstracts were reviewed by using the above mentioned selection criteria. Full papers of appropriate studies were obtained for detailed evaluation. Data extraction and quality assessment were carried out after a modified evaluation tool of the Center for Reviews and Dissemination [24]. Data extraction of *study characteristics* comprised the following data: number of included patients, number of treated patients in each group, control treatment, follow-up time, treatment regimen and dosages, baseline visual acuity, and time between symptoms and/ or diagnosis of macular edema and treatment in months. Additionally, the percentage of patients included within 3 months of disease onset was noted, if reported. If duration was given in weeks or days, values were transformed to mean time in months. Visual acuity is given in number of letters (according to ETDRS scores) and was changed to letters, if reported in logMAR. Data extraction of *study outcome* comprised, if available, the following data: number of included patients, mean number of intravitreal injections within 12 months (or from month 12 to 24), percentage of patients with gain or loss of visual acuity ≥ 15 letters, and mean change in visual acuity from baseline to month 12 (from month 12 to 24) in letters. Despite the multitude of information modi provided, we extracted information on the percentage (and/or number of patients) of endophthalmitis, uveitis, retinal detachment, retinal tear, and traumatic lens damage, rise of intraocular pressure (IOP), medical treatment of IOP rise, glaucoma surgery, cataract progression and surgery, and vitreous hemorrhage. Given the underlying disease of BRVO or CRVO, we collected also all information on vitreous hemorrhage, neovascularization of the iris (NVI) and neovascular glaucoma (NVG), if reported. Regarding systemic adverse events we extracted information on death (any cause), myocardial infarction, cerebrovascular accidents, nonocular hemorrhage and infection if obtainable. *Parameters to assess the methodological quality* of RCTs and estimate risk of bias were information on blinding, transparency of patient flow, sample size calculation, intention-to-treat analysis, definition of expected adverse events, the method used to collect adverse events data, and comparability between groups at inclusion. All stages of study selection, data extraction and quality assessment were done independently by two reviewers (AP, CI or NF). Any disagreement was resolved by discussion and consensus.

### Statistical Analysis

A narrative summary is provided because the identified studies are enormous heterogeneous in terms of intervention and sample size and, therefore, unsuitable for pooling.

## Results

### Study selection/ Included studies

**Figure 1:**
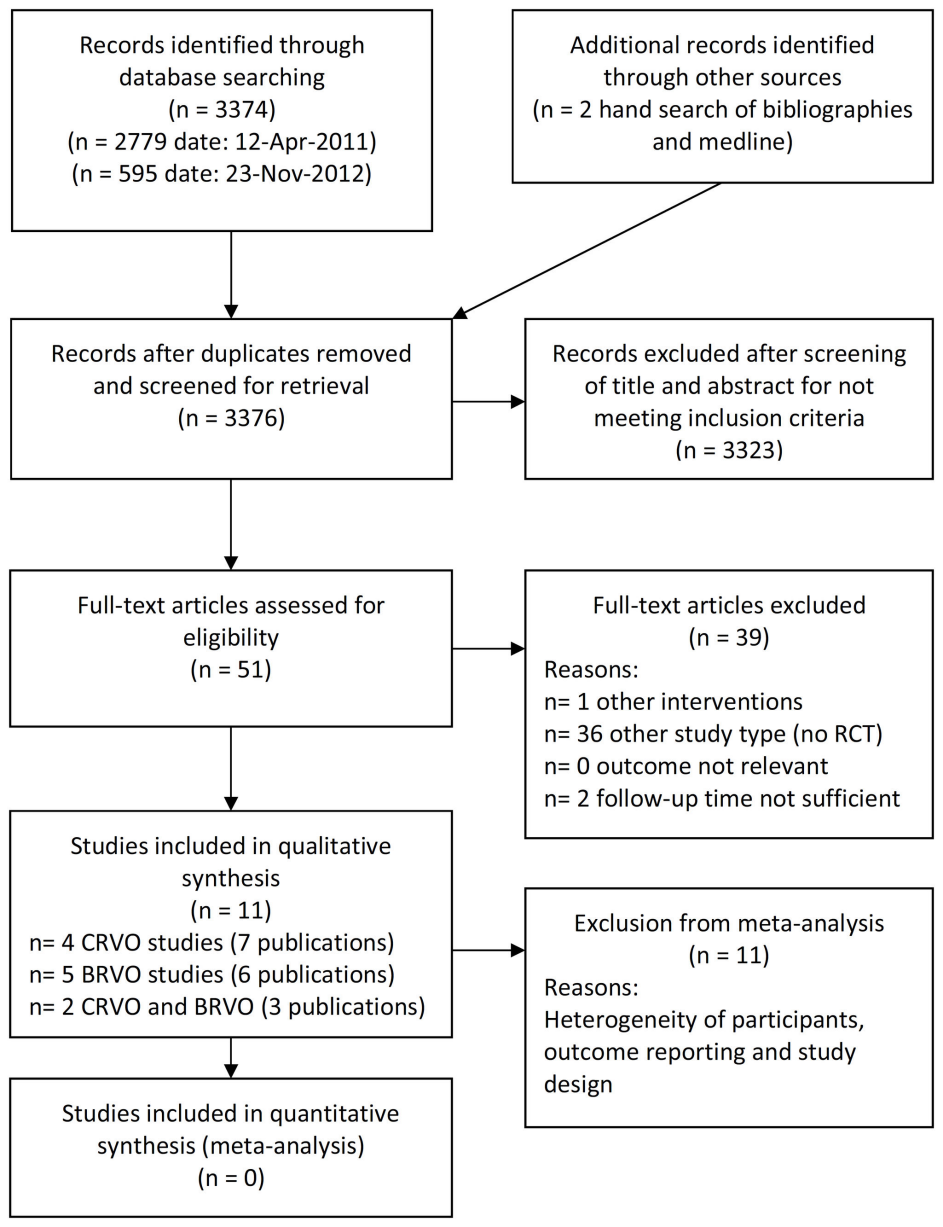
Flow chart of literature search and study selection.

After removing duplicate references, the searches identified 3374 citations (Medline (1035), Premedline (44), Medline in process (45), Cochrane Library (81) und Embase (2169) (Figure 1) modified from [25]). Of the 3374 citations, 7 RCTs were included to evaluate BRVO and 5 RCTs were included for CRVO.

### Study characteristics (Table 1)

**Table 1:** Study characteristics.

	**Study**	**Investigational product**	**Included patients**	**Treated patients**		**Control treatment**	**Follow-up (months)**	**Treatment regime**	Dosage (mg)*	**Time between symptoms/ diagnosis and treatment (months)**				**Baseline BCVA (letters)**			
				**1 mg**	**4 mg**	**Observation**				**1 mg**	**4 mg**		**Obs.**	**1 mg**	**4 mg**		**Obs.**
**CRVO**	**SCORE 2009, 2011**[5,32]	Triamcinolone	271 (81 at 3 years)	91	91	88	12 (36)	PRN every 4 months	1 and 4 (Trivaris)	4.5	4.2		4.2	51	51		52
				**0.7 mg**	**0.35 mg**	**Sham/ 0.7 mg**											
**CRVO/ BRVO**	**GENEVA 2011** [8,9]	Dexamethasone implant	1256	421	412	423	12	every 6 months	0.35 and 0.7 implant/ Sham injection	5.2 (15-18% < 3)				54			
	CRVO#			31.9%	37.2%	34.5%											
	BRVO#			68.1%	62.8%	65.5%											
				**1.25 mg**		**Sham/ 1.25 mg**				**1.25 mg**		**Sham/ 1.25 mg**		**1.25 mg**		**Sham/ 1.25 mg**	
**CRVO**	**Epstein 2012** [26]	Bevacizumab	60	30		30	12	1.25 mg/ 6 weeks or Sham/ 6 weeks + 1.25 mg/ 6 weeks	1.25	1.9		2.2		44.4		43.9	
				**2 mg**		**Sham/ 2 mg**				**2 mg**		**Sham/ 2 mg**		**2 mg**		**Sham/ 2 mg**	
**CRVO**	**COPERNICUS 2013** [20,36]	Aflibercept	189	115		74	12	Monthly for 6 months then PRN	2	2.73 (56.1% ≤2)		1.88 (71.2% ≤2)		50.7		48.9	
				**0.3/ 0.5 mg**	**0.5/ 0.5 mg**	**Sham/ 0.5 mg**											
**CRVO**	**CRUISE 2011** [22]	Ranibizumab	392	132	130	130	12	Monthly for 6 months then PRN	0.3 and 0.5	3.3 (69% ≤ 3)				48.3			
**CRVO/ BRVO**	**HORIZON 2012** [30]	Ranibizumab	181 of 304	70 of 107	51 of 99	60 of 98	12 - 24	PRN every 1-3 months	0.5	(idem CRUISE)				(idem CRUISE)			
				**0.3/ 0.5 mg**	**0.5/ 0.5 mg**	**Sham/ 0.5 mg (rescue: grid)**											
**BRVO**	**BRAVO 2011** [23]	Ranibizumab	397	134	131	132	12	Monthly for 6 months then PRN	0.3 and 0.5	3.5 (65% ≤ 3)				54.6			
**BRVO/ CRVO**	**HORIZON 2012** [30]	Ranibizumab	205 of 304	66 of 103	73 of 104	66 of 97	12- 24	PRN every 1-3 months	0.5	(idem BRAVO)				(idem BRAVO)			
				**1 mg**	**4 mg**	**Grid**				**1 mg**	**4 mg**		**Grid**				
**BRVO**	**SCORE 2009, 2011** [4,32]	Triamcinolone	411 (128 at 3 years)	136	138	137	12 (36)	PRN every 4 months	1 and 4 (Trivaris)	4.5 (38% ≤ 3)	4.6 (37% ≤ 3)		4.5 (36% ≤ 3)	57			
				**SGLT + 4 mg**		**SGLT + sham**											
**BRVO**	**Parodi 2008** [27]	Triamcinolone	24	11		13	12	4 mg triamcinolone + SGLT after 2 weeks or SGLT + sham injection	4 in 0.1 ml (Kenalog)	8				Nr			
				**1.25 mg**		**Grid**								**1.25 mg**		**Grid**	
**BRVO**	**Russo 2009** [28]	Bevacizumab	30	15		15	12	PRN	1.25	Nr				41.5		40.5	
				**1.25 mg**		**1.25 mg + grid**								**1.25 mg**		**1.25 mg + grid**	
**BRVO**	**Donati 2012** [29]	Bevacizumab	18	9		9	12	Monthly for 3 then PRN at month 3 and 6	1.25	3.17				50		55	

Nr: Not reported, PRN: pro re nata, Obs.: Observation, SGLT: subthreshold grid laser photocoagulation

^*^ Dosage in mg per 0.05 ml volume if not indicated separately

^#^ Percentage of CRVO/ BRVO patients at baseline of core-study [9]

Inclusion criteria were met by 7 multicenter RCTs and 4 single-center RCTs investigating CRVO alone (n= 4, [5,20,22,26]), BRVO alone (n= 5, [4,23,27–29]) or both CRVO/BRVO (n= 2, [8,30]). Table 1 presents study characteristics of all identified RCTs evaluating monotherapy of intravitreal triamcinolone, dexamethasone, ranibizumab, bevacizumab or aflibercept in macular edema due to CRVO or BRVO separately with a follow-up of at least 1 year. Control treatment consisted of sham injections or observation in CRVO patients (SCORE-CRVO, GENEVA, CRUISE, Epstein, and COPERNICUS [5,8,20,22,26]). In BRVO control treatment differed among the studies between sham treatment (GENEVA, BRAVO [8,23]), grid laser photocoagulation (SCORE, Parodi, Russo [4,21,22]), and grid laser photocoagulation with bevacizumab (Donati [29]). 

#### Triamcinolone - The SCORE study (CRVO/ BRVO) and Parodi 2008 (BRVO)

The SCORE (Standard Care vs. Corticosteroid for Retinal Vein Occlusion) study compared intravitreal triamcinolone (Trivaris, Allergan) 1 mg or 4 mg with observation in CRVO [5] and with grid laser photocoagulation in BRVO [4]. In total, 271 patients were included in SCORE-CRVO and longest follow-up was 36 months. In the parallel SCORE-BRVO trial 411 patients were enrolled [4]. Treatment regimen allowed re-treatment with triamcinolone at PRN criteria using 4 months intervals [4,5]. The mean time from symptoms (or diagnosis) of CRVO to treatment initiation was 4 months, only 39% of the patients were treated within 3 months and 81% within 6 months of onset [5]. Correspondingly, the time span to treatment initiation in SCORE-BRVO was 4 months [4]. 

Parodi et al. investigated subthreshold grid laser photocoagulation (SGLT) in combination with triamcinolone 4 mg (Kenalog, 40 mg/mL, Bristol-Myers Squibb, Princeton, NJ) versus SGLT and sham injection in 24 patients with BRVO [27]. Follow-up was 12 months. Mean time from symptoms to treatment was 8 months (range 3-18) [27].

#### Dexamethasone Implant - The GENEVA study (CRVO/ BRVO)

The GENEVA (Global Evaluation of Implantable Dexamethasone in Retinal Vein Occlusion With Macular Edema) study evaluated the safety and efficacy of an intravitreal dexamethasone implant 0.7 mg versus an implant 0.35 mg versus sham injection in both BRVO and CRVO. The primary outcome measure was time to visual improvement of 15 letters or more. This outcome was pooled for BRVO and CRVO patients [8]. GENEVA included 1256 patients in their open-label 6-month extension of which 1131 completed the trial. In total, 34.5% of patients presented with CRVO and 65.5% with BRVO in the GENEVA study. Time from symptoms to treatment was 5.2 months, 15-18% of patients received treatment within 3 months of diagnosis.

#### Ranibizumab - The CRUISE (CRVO) and BRAVO (BRVO) studies

The CRUISE (Ranibizumab for the Treatment of Macular Edema After Central Retinal Vein Occlusion Study: Evaluation of Efficacy and Safety) study enrolled 392 patients and assigned them either to ranibizumab 0.3 mg or 0.5 mg monthly for 6 months followed by PRN treatment of ranibizumab 0.5 mg in all groups from month 6 to 12 [22]. Control treatment in this study consisted of sham injections until month 6. Mean duration of symptoms to treatment was 3.3 months. In contrast to SCORE, the percentage of patients receiving treatment within 3 months of CRVO onset was considerably higher (69% in CRUISE versus 39% in SCORE [5,22]).

The BRAVO (Ranibizumab for the Treatment of Macular Edema Following Branch Retinal Vein Occlusion: Evaluation of Efficacy and Safety) study assigned 397 patients to 3 treatment arms (corresponding to CRUISE): intravitreal ranibizumab 0.3 mg, intravitreal ranibizumab 0.5 mg and sham injections for 6 months followed by a PRN of ranibizumab 0.5 mg for all patients until month 12 [23]. Grid laser photocoagulation was only allowed as rescue therapy (to all groups), but not earlier than 3 months after inclusion [23]. 65% of BRVO patients were treated within 3 months of RVO duration, mean time from symptoms to treatment was 3.5 months [23]. 

#### Ranibizumab in the 2^nd^ year - The HORIZON trial (BRVO and CRVO)

In cohort 2 of the open-label, single-arm, multicenter extension HORIZON trial, patients from BRAVO and CRUISE studies were included [30]. 304 patients of each trial continued treatment of macular edema with intravitreal ranibizumab 0.5 mg at PRN intervals (between 1 and 3 months) for additional 12 months. Grid laser photocoagulation was eligible as rescue therapy for BRVO patients. The HORIZON trial was discontinued after FDA approval for ranibizumab in RVO (in accordance to the study protocol). Finally, 181 of 304 CRVO patients and 205 of 304 BRVO patients were analysed. Regarding the safety analysis, data of all patients over the entire study duration of 24 months were included [30]. 

#### Bevacizumab – Epstein 2012 (CRVO), Russo 2009 and Donati 2012 (BRVO)

Intravitreal bevacizumab in CRVO was evaluated in the RCT by Epstein and colleagues [26,31]. Patients received either intravitreal bevacizumab 1.25 mg or sham injection every 6 weeks over a period of 6 months. After this, an open label extension followed during which all patients received bevacizumab 1.25 mg every 6 weeks. Each group comprised 30 patients, who showed symptoms of CRVO for 1.9 months in the bevacizumab and 2.2 months in the sham/ bevacizumab group.

Russo et al. compared intravitreal bevacizumab 1.25 mg after PRN schemata versus grid laser photocoagulation in 30 patients with BRVO over 12 months [28]. Inclusion criterion was duration of macular edema of at least 3 months. 

Donati et al. randomized 18 patients (1:1) to 3 intravitreal injections of bevacizumab 1.25 mg or intravitreal injections combined with grid laser photocoagulation (one week after first injection) [29]. Patients could receive further injections PRN. Follow-up visits were conducted at month 3, 6 and 12 (final visit). Thus the maximum number of injections per group was 5. Median duration of macular edema before treatment was 3.2 and 3.1 months, respectively [29].

#### Aflibercept - The COPERNICUS trial (CRVO)

Only recently, aflibercept has been investigated in patients with CRVO in the COPERNICUS (Controlled Phase 3 Evaluation of Repeated Intravitreal Administration of VEGF Trap-Eye in Central Retinal Vein Occlusion: Utility and Safety) trial [20]. 189 patients with CRVO were included and received intravitreal aflibercept 2 mg (aflibercept group) compared to sham treatment (sham/aflibercept group) at a randomisation ratio of 2:1 during the first 6 months followed by (monthly) PRN treatment of intravitreal aflibercept 2 mg in all patients. Primary endpoint was the proportion of patients with an improvement of BCVA ≥ 15 letters. Mean time from symptoms to treatment was relatively short with 2.7 months (aflibercept) and 1.9 months (sham/aflibercept). First treatment within 2 months occurred in 56.1% (aflibercept) and 71.2% (sham/aflibercept), respectively [20].

### Study results: Efficacy Outcome (Table 2, Figure 2, 3)

**Table 2:** Efficacy parameters.

	**Study**	**Investigational product**	**Included patients**	**Injections per patient (mean number/ 12 months)**				**Percentage of patients with BCVA gain ≥ 15 letters (at 12 months)**				**Change in BCVA at month 12 (mean number of letters)**				**Percentage of patients with BCVA loss ≥ 15 letters**			
				**1 mg**	**4 mg**		**Observation**	**1 mg**	**4 mg**		**Observation**	**1 mg**	**4 mg**		**Observation**	**1 mg**	**4 mg**		**Observation**
**CRVO**	**SCORE 2009, 2011** [5,32]	Triamcinolone	271	2.2	2.0		0.1	26.5 %	25.6%		6.8%	-1.2	-1.2		-12.1	25% (31% 24 mo)	26% (26% 24 mo)		44% (48% 24 mo)
				**0.7/ 0.7 mg**	**0.35/ 0.7 mg**		**Sham/ 0.7 mg**												
**CRVO/ BRVO**	**GENEVA 2011** [8,9]	Dexamethasone implant	1256#	1.86	1.85		0.83	Nr				Nr				Nr			
												**0.7/ 0.7 mg**		**Sham/ 0.7 mg**					
**Subgroup CRVO**	**GENEVA 2011** [8]	Dexamethasone implant	437 (at baseline)	Nr				Nr				+2 (approx.)		-1 (approx.)		Nr			
												**0.7/ 0.7 mg**		**Sham/ 0.7 mg**					
**Subgroup BRVO**	**GENEVA 2011** [8]	Dexamethasone implant	830 (at baseline)	Nr				Nr				+6 (approx.)		+6 (approx.)		Nr			
				**1.25 mg**		**Sham/ 1.25 mg**		**1.25 mg**		**Sham/ 1.25 mg**		**1.25 mg**		**Sham/ 1.25 mg**		**1.25 mg**		**Sham/ 1.25 mg**	
**CRVO**	**Epstein 2012** [26]	Bevacizumab	60	8		4/ 4		60%		33.3%		+16.1		+4.6		6.7%		6.7%	
				**2 mg**		**Sham/ 2 mg**		**2 mg**		**Sham/ 2 mg**		**2 mg**		**Sham/ 2 mg**		**2 mg**		**Sham/ 2 mg**	
**CRVO**	**COPERNICUS 2013** [20]	Aflibercept	189	8.5		5.3 / 3.9		55.3%		30.1%		+16.2		+3.8		5.3%		15.1%	
				**0.3/ 0.5 mg**	**0.5/ 0.5 mg**		**Sham/ 0.5 mg**	**0.3/ 0.5 mg**	**0.5/ 0.5 mg**		**Sham/ 0.5 mg**	**0.3/ 0.5 mg**	**0.5/ 0.5 mg**		**Sham/ 0.5 mg**	**0.3/ 0.5 mg**	**0.5/ 0.5 mg**		**Sham/ 0.5 mg**
**CRVO**	**CRUISE 2011** [22]	Ranibizumab	392	9.6	8.8		5.4/ 3.7	47%	50.8%		33.1%	+13.9	+13.9		+7.3	3.8%	2.3%		10%
**CRVO**	**HORIZON 2012** [30]	Ranibizumab	181/ 304	3.8 (12-24)	3.5 (12-24)		2.9 (12-24)	38.6% (from baseline)	45.1%		38.3%	-5.2 (12-24), +8.2 (0-24)	-4.1 (12-24), +12 (0-24)		-4.1 (12-24), +7.6 (0-24)	12.9%	5.9%		13.3%
**BRVO**	**BRAVO 2011** [23]	Ranibizumab	397	8.3	8.4		5.7/ 3.6	56%	60.3%		43.9%	+16.4	+18.3		+12.1	0%	1.5%		4.5%
**BRVO**	**HORIZON 2012** [30]	Ranibizumab	205/ 304	2.4 (12-24)	2.1		2	50% (from baseline)	60.3%		51.5%	-2.3 (12-24), +14.9 (0-24)	-0.7 (12-24), +17.5 (0-24)		+0.9 (12-24), +15.6 (0-24)	1.5%	1.4%		1.5%
				**1 mg**	**4 mg**		**Grid**	**1 mg**	**4 mg**		**Grid**	**1 mg**	**4 mg**		**Grid**	**1 mg**	**4 mg**		**Grid**
**BRVO**	**SCORE 2009, 2011** [4,32]	Triamcinolone	411 (128)	2.2	2.1		0.7 (+ H)*, 1.8 (- H)*	26%	27%		29%	+5.7	+4		+4.2	12%	12%		15%
				**4 mg + SGLT**		**SGLT**		**4 mg + SGLT**		**SGLT**		**4 mg + SGLT**		**SGLT**		**4 mg + SGLT**		**SGLT**	
**BRVO**	**Parodi 2008** [27]	Triamcinolone	24	1		1		54%		38%		+17		+6.5		0%		15%	
				**1.25 mg**		**Grid**		**1.25 mg**		**Grid**		**1.25 mg**		**Grid**		**1.25 mg**		**Grid**	
**BRVO**	**Russo 2009** [28]	Bevacizumab	30	1.7		1.5		80%		53%		+15.5		+10		Nr		Nr	
				**1.25 mg**		**1.25 mg + grid**		**1.25 mg**		**1.25 mg + grid**		**1.25 mg**		**1.25 mg + grid**		**1.25 mg**		**1.25 mg + grid**	
**BRVO**	**Donati 2012** [29]	Bevacizumab	18	4 (median)		3 (median)		33.3%		77.8%		+15		+20		0%		0%	

Nr: Not reported, SGLT: subthreshold grid lasercoagulation

* SCORE definition of two groups within the grid treatment arm: (+ H) = dense macular hemorrhages at baseline, (- H) = no hemorrhages at baseline

^#^ Patients per treatment groups in the Geneva trial: 330 0.7/ 0.7 mg, 316 0.35/ 0.7 mg, 313 sham/ 0.7 mg, 53 0.7 mg/ none, 57 0.35 mg/ none, 62 sham/ none

**Figure 2:**
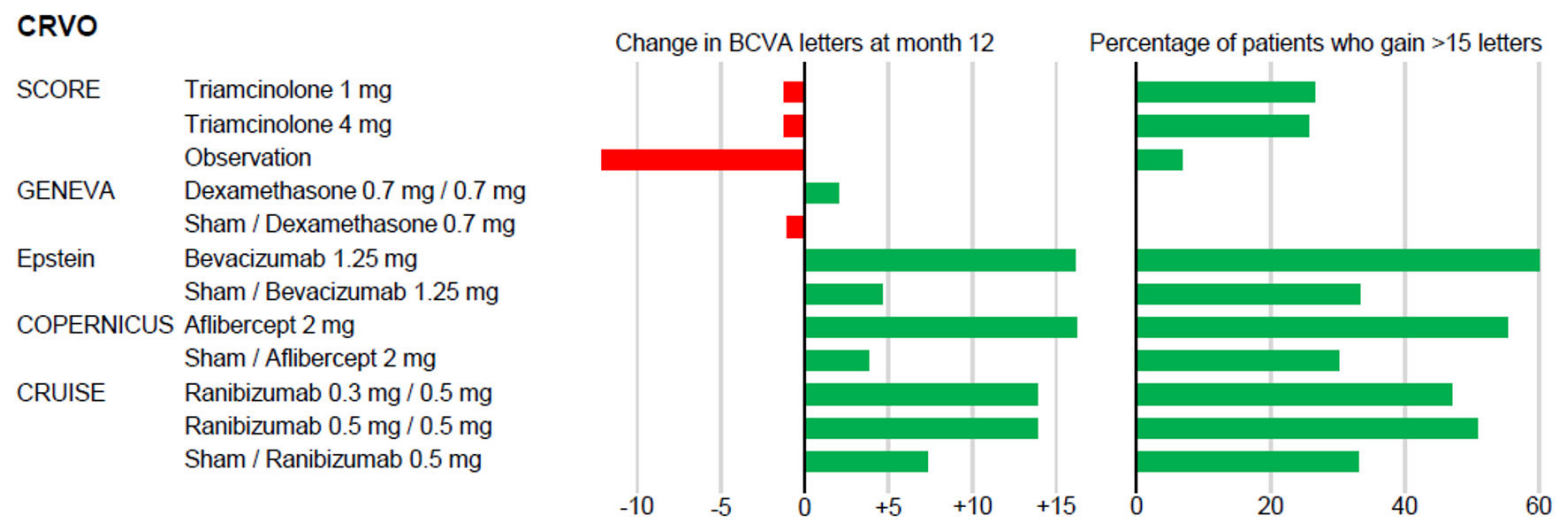
Bar graph for results in central retinal vein occlusion (CRVO). Change in best corrected visual acuity (BCVA) in letters at month 12 for each treatment group (left) and percentage of patients with gain of BCVA of equal or more than 15 letters (right).

**Figure 3:**
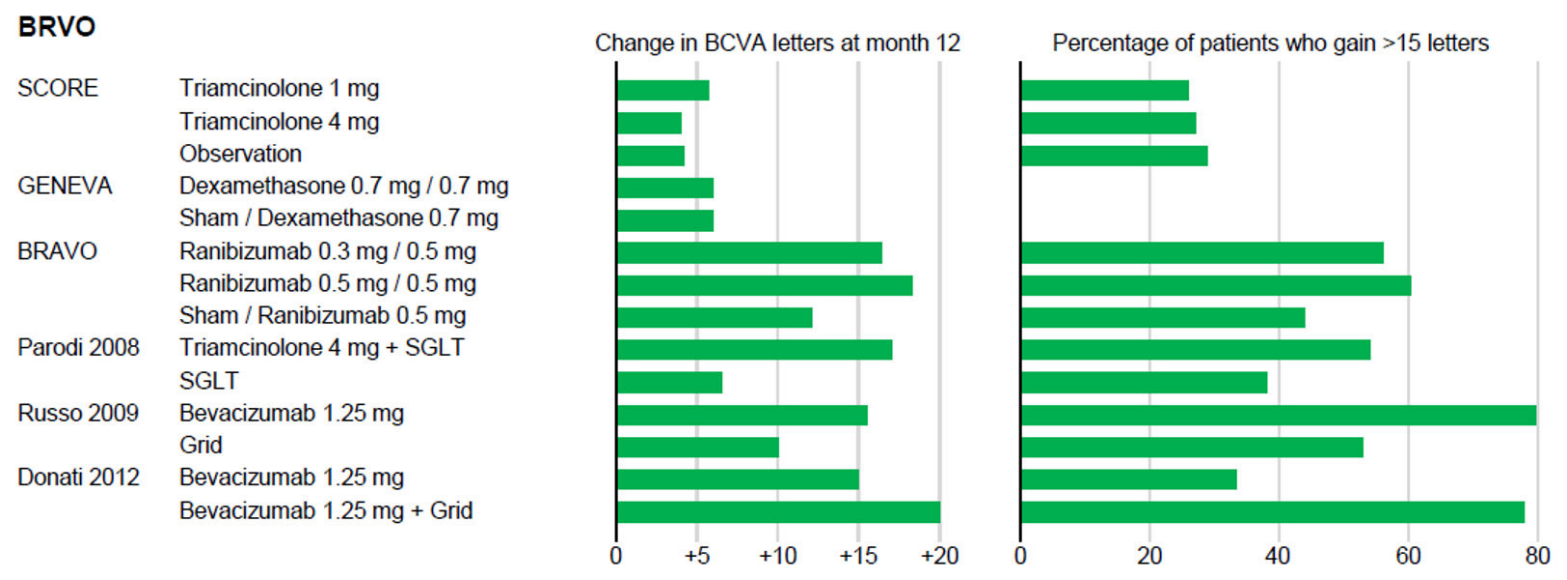
Bar graph for results in branch retinal vein occlusion (BRVO). Change in best corrected visual acuity (BCVA) in letters at month 12 for each treatment group (left) and percentage of patients with gain of BCVA of equal or more than 15 letters (right); SGLT: subthreshold grid laser photocoagulation.

Systematic extraction of outcome measures was limited by different primary outcome measures reported in the studies. 

#### Triamcinolone - The SCORE study (CRVO/ BRVO) and Parodi 2008 (BRVO)

The SCORE-CRVO study found a significant difference between triamcinolone and sham: The percentage of participants with a gain of visual acuity of 15 letters or more was 26.5%, 25.6% and 6.8% for triamcinolone 1 mg, 4 mg and sham, respectively (p=0.001 for 1 mg versus observation and also for 4 mg versus observation) [5]. Both triamcinolone concentrations stabilized visual acuity at month 12 (-1.2 letters), whereas sham injections led to a mean deterioration of -12.1 letters [5]. The percentage of participants who lost 15 letters or more was nearly doubled in the sham group (44% at month 12) compared to 25% (1 mg) and 26% (4 mg) in the triamcinolone groups [5]. This remained almost unchanged until 24 months (48% [sham] versus 31% [1 mg] and 26% [4 mg]). Mean number of injections was 2.2 (triamcinolone 1 mg), 2.0 (triamcinolone 4 mg) and 0.1 (observation). 

The SCORE-BRVO study did not find a statistically significant difference of visual acuity gain after 12 months: 26% (triamcinolone 1 mg), 27% (4 mg) and 29% (grid laser photocoagulation) [4]. Mean change in BCVA was +4 to +5 letters in all groups. It was achieved with a mean treatment application of 2.2 injections (for triamcinolone 1 mg), 2.1 injections (for triamcinolone 4 mg), and 1.8 (for grid laser photocoagulations, if no dense macular hemorrhages were present at baseline; 0.7 applications for the group, that received deferred laser after resorption of macular hemorrhages) [4]. 

Parodi et al. found a visual acuity gain of more than 15 letters in 54% of the combination group (SGLT and triamcinolone) and 38% in controls (SGLT with sham injection) after a single treatment [27]. Mean visual acuity increased by +17 letters (intervention group) and +6.5 letters (control group), respectively (p=0.011 for mean change in visual acuity at month 12 compared to baseline in the combination group).

#### Dexamethasone Implant - The GENEVA study (CRVO/ BRVO)

Primary outcome of the GENEVA open-label extension study until month 12 was safety [8]. Efficacy within the core study was measured in time to achieve a significant improvement (gain of more than 15 letters). Patients treated with a dexamethasone implant reached this goal significantly faster and in a higher percentage than sham treated patients (pooled data from BRVO and CRVO, p<0.001) [8,9]. Looking into BRVO and CRVO data separately, the GENEVA study did not find a significant difference in visual acuity at month 12 between the groups. Unfortunately, numbers are not provided within the publication, but figures indicate that change in BCVA was +2 letters in dexamethasone 0.7 mg/0.7 mg versus -1 letter in sham/0.7 mg in CRVO and +6 letters in BRVO in both groups [8]. Mean number of injections was 1.9 (0.7 mg/0.7 mg), 1.9 (0.35 mg/0.7 mg), and 0.8 (sham/0.7 mg). 

#### Ranibizumab - The CRUISE (CRVO), BRAVO (BRVO) and HORIZON trial (CRVO/ BRVO)

The CRUISE study selected the mean change from baseline BCVA letter score at month 12 as primary outcome, and also reported all parameters chosen for extraction within this search [22]. Patients treated with ranibizumab gained +13.9 letters (both groups 0.3/0.5 mg and 0.5/0.5 mg independently), and +7.3 letters, if treated 6 months with sham injections followed by ranibizumab 0.5 mg (p<0.001 for each ranibizumab group versus sham/0.5 mg group) [22]. Percentage of patients with a gain of 15 letters or more was highest in ranibizumab 0.5/0.5 mg (50.8%), versus 0.3/0.5 mg (47.0%) and sham/0.5 mg (33.1%). These gains were achieved by 9.6 (0.3/0.5 mg), 8.8 (0.5/0.5 mg), and 9.1 (sham/0.5 group [5.4 sham injections and 3.7 IVR injections]) injections after month 12 [22]. 

Results of the BRAVO trial found a mean change in BCVA of +16.4 letters (0.3/0.5 mg), +18.3 letters (0.5/0.5 mg) and +12.1 (sham/0.5 mg) (p<0.01 for each ranibizumab group versus sham/0.5 mg group) [27]. More patients treated with ranibizumab from the beginning gained ≥ 15 letters (56% and 60.3%) than those with sham injections followed by ranibizumab 0.5 mg (43.9%). Mean number of injections did not differ between groups (mean 8.3, 8.4 and 9.4 injections, respectively). 

For patients from BRAVO and CRUISE studies who continued from month 12 to month 24 within the HORIZON trial visual acuity decreased slightly (in CRVO) or remained stable (in BRVO) [30]. In HORIZON-CRVO mean visual acuity change was -5.2 (ranibizumab 0.3/0.5 mg), -4.1 (0.5/0.5 mg) and -4.2 (sham/0.5 mg) letters, respectively. Injections were given significantly less frequently during the second year, the mean number of injections was 3.8 (0.3/0.5 mg), 3.5 (0.5/0.5 mg) and 2.9 (sham/0.5 mg). The mean total change in BCVA from baseline to month 24 resulted in +8.2 (0.3/0.5 mg), +12 (0.5/0.5 mg), and +7.6 (sham/0.5 mg) letters.

HORIZON-BRVO: Patients with BRVO stabilized at the BCVA letter score gained at month 12. Mean BCVA change during the second year was -2.3 letters (0.3/0.5 mg), -0.7 letter (0.5/0.5 mg) and +0.9 letter (sham/0.5 mg). The mean difference in BCVA score between baseline and month 24 was +14.9 letters, +17.5 letters and +15.6 letters, respectively. Frequency of intravitreal injections was considerably lower than in the first year and even lower than in CRVO patients during year 2. Patients received 2.4 (0.3/0.5 mg), 2.1 (0.5/0.5 mg) and 2.0 (sham/0.5 mg) injections [30]. Caution: Numbers are probably that low because the HORIZON trial was terminated immediately after FDA approval of ranibizumab.

#### Bevacizumab - Epstein 2012 (CRVO), Russo 2009 and Donati 2012 (BRVO)

Epstein and colleagues found that CRVO patients treated with bevacizumab 1.25 mg every 6 weeks from baseline gained +16.1 letters, compared to +4.6 letters in those treated with sham injections followed by bevacizumab (p<0.05) [26]. The percentage of BCVA gain of more than 15 letters was 60% (bevacizumab) vs. 33.3% (sham/bevacizumab), respectively (p<0.05). 

Russo et al. found a change in BCVA of +15.5 letters in bevacizumab treated patients and +10 letters in grid laser photocoagulation (p<0.05), achieved by a mean number of 1.7 intravitreal injections of bevacizumab and 1.5 grid applications [28]. 

Donati et al. found an improvement in BCVA of +15 letters in patients treated with bevacizumab alone (median: 4 injections) compared to +20 letters in patients who received a combination of intravitreal bevacizumab (median 3 injections) and one grid laser photocoagulation (p<0.01 for visual acuity at month 12 versus baseline in each group, p=0.03 for difference between groups at month 12) [29]. Three of 9 bevacizumab patients gained more than 15 letters, in the group of patients who received combination therapy 7 of 9 patients reached this gain. 

#### Aflibercept - The COPERNICUS trial (CRVO)

CRVO patients in the aflibercept 2 mg group gained +16.2 letters at month 12, compared to +3.8 letters in the sham/aflibercept (p<0.001) [20]. The percentage of patients with a gain of more than 15 letters was 55.3% (aflicercept) and 30.1% (sham/aflibercept), respectively (p<0.001). After 5.8 aflibercept injections until month 6, visual acuity was stabilized with an additional 2.7 injections during PRN. In the control group, patients with a mean of 5.3 sham injections in the beginning received a mean of 3.9 injections of aflibercept 2 mg until month 12 [20]. 

### Study results: Safety Outcome – ocular adverse events (Table 3, 4)

**Table 3:** Ocular safety – Endophthalmitis, uveitis, retinal detachment, retinal tear, traumatic lens damage.

	**Study**	**Investigational product**	**Total number of injections**	**Endophthalmitis [%], (number of eyes)**				**Uveitis [%], (number of eyes)**			**Retinal detachment [%], (number of eyes)**			**Retinal tear [%], (number of eyes)**				**Traumatic lens damage [%], (number of eyes)**
**CRVO**	**SCORE 2009, 2011** [5,32]	Triamcinolone	586	0				0			0			Nr				Nr
**CRVO/ BRVO**	**GENEVA 2011** [8,9]	Dexamethasone implant	1715 implants#	0				Nr			Rare			Rare				Nr
**CRVO**	**Epstein 2012** [26,31]	Bevacizumab	720	0				Nr			0			0				Nr
				**2 mg**		**Sham/ 2 mg**								**2 mg**		**Sham/ 2 mg**		
**CRVO**	**COPERNICUS 2013** [20,36]	Aflibercept	1266*	0.9% (1)		0		Nr			Nr			0		1.7% (1)		Nr
				**0.3/ 0.5 mg**	**0.5/ 0.5 mg**		**Sham/ 0.5 mg**	**0.3/ 0.5 mg**	**0.5/ 0.5 mg**	**Sham/ 0.5 mg**	**0.3/ 0.5 mg**	**0.5/ 0.5 mg**	**Sham/ 0.5 mg**	**0.3/ 0.5 mg**	**0.5/ 0.5 mg**		**Sham/ 0.5 mg**	
**CRVO**	**CRUISE 2011** [22]	Ranibizumab	2892*	0				2.3% (3)	1.6% (2)	3.9% (5)/ 1.8% (2)	0			0	1.6% (2)		0/ 1.8% (2)	0
**CRVO/ BRVO**	**HORIZON 2012** [30]	Ranibizumab		0.9% (2)	0		0	0			Nr			0				Nr
**BRVO**	**BRAVO 2011** [23]	Ranibizumab	2679*	0	0.8% (1)		0	2.2% (3)	0	3.1% (4)/ 0.9% (1)	0.7% (1)	0	0	0.7% (1)	0		0	0
**BRVO/ CRVO**	**HORIZON 2012** [30]	Ranibizumab		0				0			Nr			0				Nr
				**1 mg**	**4 mg**		**Grid**				**1 mg**	**4 mg**	**Grid**					
**BRVO**	**SCORE 2009, 2011** [5,32]	Triamcinolone	914	0	0.1% (1)		0	Nr			0.7% (1)	0	0.7% (1)	Nr				Nr
**BRVO**	**Parodi 2008** [27]	Triamcinolone	11	0				0			0			Nr				0
				**1.25 mg**		**Grid**												
**BRVO**	**Russo 2009** [28]	Bevacizumab	25	0		0		0			Nr			Nr				Nr
**BRVO**	**Donati 2012** [29]	Bevacizumab	63*	Nr				Nr			Nr			Nr				Nr

Nr: Not reported

* Result of calculation as follows: median number of intravitreal injection multiplicated by number of participants per group

^#^ Number of implants by dosing groups: 1342 0.7 mg, 373 0.35 mg

**Table 4:** Ocular safety – IOP, glaucoma, cataract, vitreous hemorrhage.

	**Study**	**Investigational product**	**IOP increase [%], (number of eyes)**					**Medical treatment of IOP [%], (number of eyes)**				**Glaucoma surgery [%], (number of eyes)**						**Cataract progression [%], (number of eyes)**					**Cataract surgery [%], (number of eyes)**				**Vitreous hemorrhage [%], (number of eyes)**			
			**1 mg**	**4 mg**			**Obs.**	**1 mg**	**4 mg**		**Obs.**	**1 mg**	**4 mg**				**Obs.**	**1 mg**	**4 mg**			**Obs.**	**1 mg**	**4 mg**		**Obs.**	**1 mg**	**4 mg**		**Obs.**
**CRVO**	**SCORE 2009, 2011** [5,32]	Triamcinolone	5.4% (5)	8.8% (8)			1.1% (1)	20% (18)	35% (32)		8% (7)	2.2%	0				0	26%	33%			18%	0	4.9% (4)		0	4.3% (4)	0		4.5% (4)
	12-24 months												4% (2)										5.5% (3)	42% (21)						
			**0.7/ 0.7 mg**	**0.35/ 0.7 mg**			**No implant**	**0.7/ 0.7 mg**	**0.35/ 0.7 mg**		**No implant**	**Any implant**			**no implant**			**0.7/ 0.7 mg**	**0.35/ 0.7 mg**			**No implant**	**0.7/ 0.7 mg**	**0.35/ 0.7 mg**		**No implant**				
**CRVO/ BRVO**	**GENEVA 2011** [8,9]	Dexamethasone implant	32.8%					25.5%	25%		Nr	1.3% (14)			0			29.8%	19.8%			5.7%	1.3%	1.8%		1.1%	Nr			
	6-12 months							+10.3%	+10.3%																					
**CRVO**	**Epstein 2012** [26]	Bevacizumab	Nr					Nr				Nr						Nr					Nr				Nr			
			**2 mg**		**Sham/ 2 mg**			**2 mg**		**Sham/ 2 mg**		**2 mg**		**Sham/ 2 mg**				**2 mg**		**Sham/ 2 mg**			**2 mg**		**Sham/ 2 mg**		**2 mg**		**Sham/ 2 mg**	
**CRVO**	**COPERNICUS 2013** [20,36]	Aflibercept	12.3%		13.5%			Nr				0		2.7% (2)/ 1.7% (1)				0.9% (1)		1.7% (1)			Nr				0.9% (1)		5.4% (4)/ 1.7% (1)	
			**0.3/ 0.5 mg**	**0.5/ 0.5 mg**			**Sham/ 0.5 mg**	**0.3/ 0.5 mg**	**0.5/ 0.5 mg**		**Sham/ 0.5 mg**	**0.3/ 0.5 mg**	**0.5/ 0.5 mg**				**Sham/ 0.5 mg**	**0.3/ 0.5 mg**	**0.5/ 0.5 mg**		**Sham/ 0.5 mg**		**0.3/ 0.5 mg**	**0.5/ 0.5 mg**		**Sham/ 0.5 mg**	**0.3/ 0.5 mg**	**0.5/0.5 mg**		**Sham/ 0.5 mg**
**CRVO**	**CRUISE 2011** [22]	Ranibizumab	Nr					Nr				Nr						3.8% (5)	7% (9)		0/ 1.8% (2)		Nr				5.3% (7)	5.4% (7)		7% (9)/ 1.8% (2)
**CRVO/ BRVO**	**HORIZON 2012** [30]	Ranibizumab	0	0.9% (1)		0		Nr				Nr						5.6% (6)	5.1% (5)		3.1% (3)		Nr				0	0		1% (1)
**BRVO**	**BRAVO 2011** [23]	Ranibizumab	Nr					Nr				Nr						4.5% (6)	6.2% (8)		3.1% (4)/ 2.6% (3)		Nr				5.2% (7)	1.5% (2)		4.6% (6)/ 0.9% (1)
**BRVO/ CRVO**	**HORIZON 2012** [30]	Ranibizumab	1% (1)	0.9% (1)		0		Nr				Nr						9.7% (10)	5.8% (6)		6.5% (6)		Nr				1% (1)	0		1.1% (1)
			**1 mg**	**4 mg**			**Grid**	**1 mg**	**4 mg**		**Grid**	**1 mg**	**4 mg**			**Grid**		**1 mg**	**4 mg**		**Grid**		**1 mg**	**4 mg**		**Grid**	**1 mg**	**4 mg**		**Grid**
**BRVO**	**SCORE 2009, 2011** [4,32]	Triamcinolone	Nr					7%	41%		2%	0	0			0		25%	35%		13%		0	2.9% (4)		2.2% (3)	0.7% (1)	2.2% (3)		1.5% (2)
	12-24 months												2.8% (2)										9.5% (8)	48.6% (35)		7.3% (6)				
								**4 mg + SGLT**		**SGLT**																				
**BRVO**	**Parodi 2008** [27]	Triamcinolone	Nr					54%		0		Nr						Nr					Nr				0			
**BRVO**	**Russo 2009** [28]	Bevacizumab	0					0				0						0					0				Nr			
**BRVO**	**Donati 2012** [29]	Bevacizumab	Nr					Nr				Nr						Nr					Nr				Nr			

IOP: intraocular pressure, Nr: not reported, PRN: pro re nata, Obs.: Observation, SGLT: subthreshold grid laser photocoagulation

#### Triamcinolone - The SCORE study (CRVO/ BRVO) and Parodi 2008 (BRVO)

The SCORE CRVO found no significantly increased rate of endophthalmitis, uveitis and retinal detachment after a total of 586 intravitreal triamcinolone injections at 12 months [5]. However, IOP rise and cataract progression occurred more frequently in the triamcinolone groups than in the observation group. 20% of patients treated with triamcinolone 1 mg and 35% of patients treated with triamcinolone 4 mg received antiglaucomatous medication compared to 8% of patients included in the observation group (p=0.02 for observation versus 1 mg, p<0.001 for observation versus 4 mg, p=0.02 1 mg versus 4 mg). Glaucoma surgery was performed in 2 patients (2.2%) treated with triamcinolone 1 mg until month 12 and 2 patients (4%) treated with 4 mg between month 12 and 24 [5]. Cataract progression was seen in 26% (1 mg), 33% (4 mg) and 18% (observation) of patients (p=0.14, X^2^ test). No cataract surgery was performed in the observation group (0%), whereas continuous cataract progression led to surgery in 5.5% of patients treated with triamcinolone 1 mg until month 24. The rate of cataract surgery was considerably higher in the triamcinolone 4 mg group with 4.9% of patients until month 12, and 42% between month 12 and 24 [5]. 

SCORE-BRVO reported 1 case of endophthalmitis in 914 intravitreal injections (0.1%) [5]. Furthermore, 1 retinal detachment (0.7%) was seen in each of the grid and triamcinolone 1 mg groups. Likewise in CRVO patients, IOP rise (2-41%) and cataract progression (13-35%) were the most frequent ocular adverse events and found most frequently in patients treated with triamcinolone 4 mg. From the patients with increased IOP, 2% (grid), 7% (1 mg) and 41% (4 mg) received antiglaucomatous medication (p=0.03 for grid versus 1 mg, p<0.001 for grid versus 4 mg and also for 1 mg versus 4 mg, X^2^ test), no glaucoma surgery was performed until month 12; however, 2 patients (2.8%) within the triamcinolone 4 mg group underwent surgery until month 24 [4]. Cataract progression occurred in 13% (grid), 25% (1 mg) and 35% (4 mg) of patients (p=0.03 for grid versus 1 mg, p<0.001 for grid versus 4 mg, p=0.1 for 1 mg versus 4 mg) and led to an increasing number of cataract surgeries from year 1 to year 2 in all groups (grid: 2.2% to 7.3%, 1 mg: 0% to 9.5%, and 4 mg: 2.9% to 48.6%) [4].

Considering neovascular events, the SCORE study provided solid and detailed information on year 1 and 2 adverse events [4,5], and looked at 36-month incidences and differences between the treatment groups in an additional report [32]. Neovascularization of the iris (NVI) and neovascular glaucoma (NVG) occurred at a rate of 3.2% (NVI) and 5.8% (NVG) in CRVO (36-month over-all incidence) and at a less frequent rate in BRVO (0.3% NVI versus 2.2% NVG) [32]. The cumulative incidence at 12 and 36 months was 6.1% for NVI and 8.5% for NVG in CRVO, compared to 1.3% NVI and 2.4% NVG in BRVO [32]. The investigators did not find a significant difference between treatment groups (CRVO p=0.31, BRVO p=0.18). The 36-month incidences for preretinal hemorrhage (PRH) or vitreous hemorrhage (VH) were 7.6% in CRVO and 3.8% in BRVO. The cumulative incidences at 12 and 36 months for neovascularization of the disc or elsewhere (NVD/E) were 2.8% (CRVO) and 2.9% (BRVO) and 8.8% (CRVO) and 7.6% (BRVO) for PRH/VE, respectively [32]. 

Parodi et al. did not see any endophthalmitis in 11 patients treated with a single triamcinolone injection after 12 months [27]. The only ocular adverse event that occurred was a rise in IOP which was treated with antiglaucomatous medication in 54% of the triamcinolone plus SGLT group compared to 0% in the SGLT group [27]. 

#### Dexamethasone Implant - The GENEVA study (CRVO/ BRVO)

Safety outcomes were not stratified into CRVO and BRVO patients but comparison was possible between the 6 different treatment arms (stratified by the number and dose of dexamethasone implant) at month 12 [8]. Haller et al. reported no endophthalmitis and a “rare” occurrence of retinal detachment and retinal tear without giving any numbers. Frequently observed adverse events were IOP rise and cataract progression. The increase of IOP ≥ 10 mmHg was most pronounced at day 60 with 32.8% over all injections (12.6% after the 1st injection and 15.4% after the 2nd, dexamethasone 0.7/0.7 mg group). Taken all dexamethasone treatment groups together, 1.3% of patients underwent glaucoma surgery compared to 0.0% of patient in the sham/ no implant group. 25.5% (dexamethasone 0.7/0.7 mg), 25.0% (dexamethasone 0.35/0.7 mg), and 28.1% (sham/dexamethasone 0.7 mg) received medical treatment for IOP compared to 0% in 0.35 mg/no implant group and sham/no implant group [8]. Cataract progression varied between 5.7% (sham/no implant), 10.5% (sham/0.7 mg) and 29.8% (0.7/0.7 mg) dexamethasone treated patients. There was no safety information on vitreous hemorrhage or neovascularization [8]. 

#### Ranibizumab - CRUISE (CRVO), BRAVO (BRVO), and HORIZON trials (CRVO/ BRVO)

The incidence of endophthalmitis was 0% in all groups of the CRUISE and BRAVO trials during year 1 [22,23]. During year 2, two patients (0.9%) of the ranibizumab (0.3/0.5 mg) group suffered from endophthalmitis [30]. Retinal tears and retinal detachment were rare among all groups (rate ranged between 0.7% and 1.8%). IOP increase was not reported during the first year [22,23] and rare during the second year [30] (0.9% of each group [CRUISE 0.5/0.5 mg, BRAVO 0.3/0.5 mg and 0.5/0.5 mg] showed an increased IOP). No surgeries and no medical treatments of IOP were reported. No cataract surgery was performed and progression of cataract was low. CRUISE found 3.8% (0.3/0.5 mg ranibizumab) and 7.0% (0.5/0.5 mg) cataract progression within 1 year compared to 1.8% (sham/0.5 mg ranibizumab) [22]. BRAVO found comparable low rates of 4.5% (ranibizumab 0.3/0.5 mg), 6.2% (0.5/0.5 mg) and 5.7% (sham/0.5 mg) [23]. During the second year rates remained fairly stable [30]. 

#### Bevacizumab - Epstein 2012 (CRVO), Russo 2009 and Donati 2012 (BRVO)

Epstein et al. did not report any endophthalmitis, nor retinal tear or detachment in both groups [26]. In the sham group 16.7% developed NVI, but no one in the bevacizumab group. By month 12 no new development of NVI was found in either group. Furthermore, in all 16.7% NVI cases, neovascularization regressed completely until month 12 after treatment changed from sham to bevacizumab 1.25 mg [26]. 

Russo et al. and Donati et al. reported no ocular adverse events in 25 intravitreal injections of bevacizumab [28,29]. Neither group showed complications during grid laser photocoagulation [28]. 

#### Aflibercept - The COPERNICUS trial (CRVO)

One of 189 patients presented with infectious endophthalmitis after intravitreal aflibercept 2 mg (0.9% [aflibercept], and 0% [sham/aflibercept]) [20]. Retinal tear was rare and occurred in 1 patient (1.7%) of the sham/aflibercept group. Vitreous hemorrhage was seen in 0.9% (aflibercept) compared to 7.1% (sham/aflibercept, 5.4% at 6 months and 1.7% until 12 months). NVI was found in 2.7% and NVG in 6.8% of control patients, whereas none of the aflibercept 2 mg group showed neovascular changes. Three patients (4.4%) of the sham/aflibercept group (2.7% at 6 months and 1.7% at 12 months) underwent glaucoma surgery, but none of the aflibercept patients. Increased IOP was equally distributed between both groups (12.3% [aflibercept] and 13.5% [sham/aflibercept]), as well as cataract progression (0.9% and 1.7%, respectively) [20].

### Study results: Safety Outcome – systemic adverse events (Table 5)

**Table 5:** Systemic safety.

	**Study**	**Investigational product**	**Death (any cause) [%], (number)**					**Myocardial infarction [%], (number)**					**Cerebrovascular accident [%], (number)**					**Nonocular hemorrhage [%], (number)**					**Infections [%], (number)**			
			**1 mg**	**4 mg**			**Obs.**	**1 mg**	**4 mg**			**Obs.**	**1 mg**	**4 mg**			**Obs.**	**1 mg**	**4 mg**			**Obs.**	**1 mg**	**4 mg**		**Obs.**
**CRVO**	**SCORE 2009, 2011** [5,32]	Triamcinolone	1.1% (1)	2.2% (2)			0	Nr					Nr					Nr					15%	19%		10%
	12-24 months		1% (1)	3.4% (3)			1.1% (1)																			
**CRVO/ BRVO**	**GENEVA 2011** [8]	Dexamethasone implant	Nr					Nr					Nr					Nr					Nr			
													**1.25 mg**		**Sham/ 1.25 mg**											
**CRVO**	**Epstein 2012** [26]	Bevacizumab	Nr					Nr					0		3.3% (1)			Nr					Nr			
			**2 mg**		**Sham/ 2 mg**			**2 mg**		**Sham/ 2 mg**			**2 mg**		**Sham/ 2 mg**			**2 mg**		**Sham/ 2 mg**			**2 mg**		**Sham/ 2 mg**	
**CRVO**	**COPERNICUS 2013** [20]	Aflibercept	0		2.7% (2)			0.9% (1)		1.7% (1)			0		1.7% (1)			Nr					7.9% NPG, 7.9% URI		6.8% NPG, 5.4% URI	
			**0.3/ 0.5 mg**	**0.5/ 0.5 mg**		**Sham/ 0.5 mg**		**0.3/ 0.5 mg**	**0.5/ 0.5 mg**		**Sham/ 0.5 mg**		**0.3/ 0.5 mg**	**0.5/ 0.5 mg**		**Sham/ 0.5 mg**		**0.3/ 0.5 mg**	**0.5/ 0.5 mg**			**Sham/ 0.5 mg**	**0.3/ 0.5 mg**	**0.5/ 0.5 mg**		**Sham/ 0.5 mg**
**CRVO**	**CRUISE 2011** [22]	Ranibizumab	0	0.8% (1)		0		0.8% (1)	0.8% (1)		0.8% (1)		0.8%	1.6% #		0		0					Nr			
**CRVO/ BRVO**	**HORIZON 2012** [30]	Ranibizumab	1% (1)	3% (3)		3.1% (3)		0.9% (1)	0		0		0.9% (1)	2.1% (2)		1% (1)		0	2% (2)		2.1% (2)		7.3% (7) NPG	7.1% (7) NPG		7.3% (7) NPG
**BRVO**	**BRAVO 2011** [23]	Ranibizumab	0	0		0		0	0.8% (1)		0.9% (1)		0.7% (1)	0.8% (1)		0.8% (1)		1.5% (2)	0.8% (1)		0		Nr			
**BRVO/ CRVO**	**HORIZON 2012** [30]	Ranibizumab	1% (1)	2.9% (3)		0		1% (1)	1% (1)		0		2.9%	0		1% (1)		0	2.9% (3)		0		5.3% NPG	1% (1) NPG		7.5% (7) NPG
			**1 mg**	**4 mg**		**Grid**		**1 mg**	**4 mg**			**Grid**	**1 mg**	**4 mg**			**Grid**	**1 mg**	**4 mg**			**Grid**	**1 mg**	**4 mg**		**Grid**
**BRVO**	**SCORE 2009, 2011** [4,32]	Triamcinolone	2.2% (3)	1.4% (2)		2.1 % (3)		Nr					Nr					Nr					16%	15%		10%
	12-24 months		(2)	(7)		(2)																				
**BRVO**	**Parodi 2008** [27]	Triamcinolone	Nr					Nr					Nr					Nr					Nr			
**BRVO**	**Russo 2009** [28]	Bevacizumab	Nr					Nr					Nr					Nr					Nr			
**BRVO**	**Donati 2012** [29]	Bevacizumab	Nr					Nr					Nr					Nr					Nr			

Obs.: Observation, Nr: not reported, NPG: Nasopharyngitis, URI: upper respiratory infection

^#^ sum of one TIA (0.8%) and one ischemic stroke (0.8%)

In general, the incidence of systemic adverse events was low and did not significantly differ between treatment groups in the multicenter trials [4,5,8,20,22,23,30]. The single center trials did not report any non-ocular adverse events [27–29]. 

#### Triamcinolone - The SCORE study (CRVO/ BRVO)

In the SCORE-CRVO study 1.1% of patients (1 mg), 2.2% (4 mg) and none (observation) died within 12 months. During the second year rates of deaths remained low with 1% (1 mg), 3.4% (4 mg), and 1.1% (observation) [5]. Death originated from different causes: among cardiovascular incidences, 1 myocardial infarction (4 mg) and 1 brain hemorrhage (1 mg) led to death. Infections were equally distributed between groups (15% [1 mg], 19% [4 mg] and 10% (observation), respectively [5]. In comparison, SCORE-BRVO reported 7 deaths equally distributed between groups (Table 5) [4]. The class of infections was found most frequently among systemic adverse events, i.e. 10% (grid), 16% (1 mg) and 15% (4 mg; not significant). 

#### Dexamethasone Implant - The GENEVA study (CRVO/ BRVO)

No systemic adverse events are reported in this trial [8].

#### Ranibizumab - CRUISE (CRVO), BRAVO (BRVO) and HORIZON trial (CRVO/ BRVO)

CRUISE investigators found an incidence of non-ocular serious adverse events potentially related to VEGF inhibition of 1.6% (in patients treated with ranibizumab 0.3/0.5 mg), 3.2% (in patients treated with 0.5/0.5 mg) and 1.6% (of sham treated patients) until month 6. Systemic adverse events were not reported in the sham/0.5 mg PRN group until month 12 [22]. Death by vascular causes did not occur among all groups and death from unknown cause was found in 0.8% in the ranibizumab 0.5/0.5 mg group. One patient of each group suffered from myocardial infarction (0.8%). Cerebrovascular events were rare with 0.8% (0.3/0.5 mg), 1.6% (0.5/0.5mg), and 0% (sham/0.5 mg) [22]. Non-ocular hemorrhages did not occur and infections were not reported [22]. 

BRAVO investigators reported an incidence of non-ocular serious adverse events potentially related to VEGF inhibition of 4.5% (ranibizumab 0.3/0.5 mg), 4.6% (0.5/0.5 mg), 0.8% (sham until month 6) and 1.7% (sham/0.5 mg) until month 12 [23]. Myocardial infarction occurred in 0% (0.3/0.5 mg), 0.8% (0.5/0.5 mg) and 0.9% (sham/0.5 mg). One of each group suffered from stroke (0.7-0.8%). In 1.5% (0.3/0.5 mg), 0.8% (0.5/0.5 mg), and 0% (sham/0.5 mg) of patients non-ocular hemorrhage was reported [23]. Rates remained low during the course of year 2 (0-3%) [30]. Rates of infections, i.e. nasopharyngitis, are given for HORIZON patients and were 7% in every CRVO group, but differed among BRVO groups between 1% (0.5/0.5 mg), 5.3% (0.3/0.5 mg), and 7.5% (sham/0.5 mg) [30].

#### Bevacizumab - Epstein (CRVO)

Epstein et al. did not report serious non-ocular adverse effects [26,31]. However, one patient (3.3%) from the sham/bevacizumab group suffered from a transient ischemic attack and dropped out of the study. 

#### Aflibercept - The COPERNICUS trial (CRVO)

During the first 12 months of the ongoing COPERNICUS trial, no deaths were reported in the aflibercept 2 mg group. However, 2 deaths (2.7%) occurred in the sham/aflibercept group [20]. Both deaths had a vascular cause (myocardial infarction and cardiac arrhythmia). Non-lethal myocardial infarction was seen in 1 patient of each group (0.9% and 1.7%). The most frequent infections were nasopharyngitis (7.9% [aflibercept] and 6.8% [sham/aflibercept]) and upper respiratory infections (7.9% versus 5.4%). Overall non-ocular serious adverse events were rare and similarly distributed between groups (5.3% [aflibercept] versus 8.1% [sham] at six months, and 6.4% [aflibercept] versus 8.3% [sham/aflibercept] at 12 months) [20]. The incidence of systemic adverse events did not differ significantly between groups.

### Methodological Quality and Risk of Bias (Table 6)

**Table 6:** Methodological Quality and Risk of Bias.

	**Study**	**adequate blinding**	**transparency of patient flow**	**Comparability between groups**	**sample size calculation**	**ITT-analysis**	Validity efficacy**	**definition of expected AE**	**definition of method used to collect AE data**	Validity safety***
**CRVO**	**SCORE 2009, 2011** [5,32]	No *	Yes	Yes	Yes	Yes	High	Yes	Yes	High
**BRVO**	**SCORE 2009, 2011** [4,32]	No *	Yes	Yes	Yes	Yes	High	Yes	Yes	High
**CRVO/ BRVO**	**GENEVA 2011** [8,9]	No ##	Yes	Yes	No	unclear +	Moderate - low	Yes	Yes	High
**CRVO**	**Epstein 2012** [26,31]	No ##	Yes	Yes	Yes	Yes	High	No	Yes	Moderate
**CRVO**	**COPERNICUS 2013** [20,36]	Yes	Yes	Yes	Yes	Yes	High	Yes	Yes	High
**CRVO**	**CRUISE 2011** [22]	Yes	Yes	Yes	Yes $	Yes	High	No	Yes	Moderate
**BRVO**	**BRAVO 2011** [23]	Yes	Yes	Yes	Yes $	Yes	High	No	No	moderate
**CRVO/ BRVO**	**HORIZON 2012** [30]	No	Yes	Yes	No	Yes	Moderate	No	Yes	Moderate
**BRVO**	**Parodi 2008** [27]	Unclear §	Yes ++	Yes	No	Unclear	Low	No	No	Low
**BRVO**	**Russo 2009** [28]	No	Yes ++	Yes	No	Unclear	Low	No	No	Low
**BRVO**	**Donati 2012** [29] **#**	No	Yes ++	Yes	No	Unclear	Low	No	No	Low

IIT: Intention-to-treat, AE: adverse event

^#^ Studies with investigational product in treatment and control group (i.e. control = combination therapy)

** Validity of efficacy is based on: adequate blinding, transparency of patient flow, comparability between groups, sample size calculation and ITT-analysis

*** Validity of safety is based on: definition of expected AE, definition of method used to collect AE data, blinding, transparency of patient flow, and comparablity between groups

* Blind to dosage of triamcinolone (1 mg or 4mg), but not to treatment arms (observation versus triamcinolone)

^##^ Double-masked trial for 6 months, followed by open-label for 6 months

^§^ patient blind regarding intravitreal injection (ranibizumab or sham), unclear, if treating and examiners blind

$ Study was not powered to compare efficacy outcomes at 6 months, efficacy analysis based on descriptive statistics, 12 months data post hoc analysis

^+^ Results of the 2 trials were pooled, results in the open label extension were analyzed for all patients according to the actual treatment received

^++^ description of all patients all visits within results, but no comment within statistics

Adequate blinding over 12 months was reported in 3 trials (COPERNICUS, CRUISE, BRAVO) [20,22,23]. SCORE investigators were blind to the dosage (triamcinolone 1 mg versus 4 mg), but not to observation [4,5]. Two studies changed the status of blinding after the primary end point was reached from double-blind to open-label at month 6 (GENEVA, Epstein) [8,26]. HORIZON followed patients from CRUISE and BRAVO trials in an open-label design after month 12 [30]. However, interpretation of data is limited by the high drop-out rate in all groups and the early termination after approval for ranibizumab. Validity of efficacy was high in 6 trials based on adequate blinding, transparency of patient flow, comparability between groups, sample size calculation and ITT-analysis (SCORE-BRVO/ -CRVO, Epstein, COPERNICUS, CRUISE and BRAVO). 

Based on our quality assessment, validity of safety was high in 4 trials (SCORE-BRVO/ -CRVO, GENEVA, and COPERNICUS) [4,5,8,20]. Four other trials showed moderate validity due to incomplete definition of expected adverse events or methods used to collect data on adverse events (Epstein, CRUISE, BRAVO, HORIZON) [22,23,26,30]. Information on ocular and systemic adverse events varied between very detailed reporting within most multicenter trials (including additional information available as online supplements) and, on the other hand, the overall notification of “no ocular and systemic adverse events” in a single-center trial. 

The trials by Parodi et al., Russo et al. and Donati et al. substantially lack quality (unclear or no blinding, moderate (low) transparency of patient flow, lack of information on sample size calculation, data analysis, adverse events collection and reporting) [27–29]. Additionally, comparability of groups is impaired (due to small sample size) [27–29].

## Discussion

### Principal Findings

#### Visual acuity

All anti-VEGF agents show a better gain of BCVA than steroids at month 12 (Figure 2 CRVO, 3 BRVO). A significant improvement of BCVA after 12 months of intravitreal anti-edematous therapy in CRVO is achieved with aflibercept 2 mg every 4 weeks for 6 months followed by a monthly PRN scheme (+16.2 letters, 8.5 injections) as well as continuous intravitreal injections of bevacizumab 1.25 mg every 6 weeks (+16.1 letters, 8 injections) [20,26]. In addition, ranibizumab (0.5 mg) leads to an improvement of VA of +13.9 letters in fixed plus monthly PRN dosage regimen as shown in the CRUISE study [22]. 

The SCORE-CRVO and GENEVA studies resulted in a stabilization of BCVA at month 12 with considerable less frequent injections (SCORE: -1.2 letters [2 injections], GENEVA: approximately +2 letters [1.8 injections]) [5,8]. Improvement of BCVA in triamcinolone treated patients was significantly better than in the observation group [5]. 

In BRVO best improvement of BCVA was found for ranibizumab in BRAVO at month 12 [23]. Treatment with 0.5 mg ranibizumab monthly for 6 months followed by monthly PRN resulted in +18.3 letters (8.4 injections) [23]. In comparison, bevacizumab seems to achieve similar results (+15 to 17 letters), but interpretation is limited by the very low numbers of participants in the included RCTs (9 to 15 patients per group) [27–29]. The RCT for aflibercept in BRVO is not yet published (“Study to Assess the Clinical Efficacy and Safety of VEGF Trap-Eye (Intravitreal Aflibercept Injection [IAI]), Also Commercially Known as EYLEA™ in Patients With Branch Retinal Vein Occlusion (BRVO)”, NCT01521559, Regeneron Pharmaceuticals, source: clinicaltrials.gov). A multitude of non-RCTs is published on bevacizumab in RVO, but lack of quality, especially in adverse events documentation and reporting, low numbers of participants and multitude of therapeutic strategies lead to exclusion within this review. 

BCVA results at 12 months were less favorable for steroids in BRVO. No significant effect was found for triamcinolone in SCORE-BRVO versus grid (2.1 injections) [4]. BRVO patients treated with dexamethasone implant deteriorated to approximately +6 letters after temporary visual improvement at month 12 (1.8 injections) [8].

#### Grid laser photocoagulation

Despite the gold standard of grid laser photocoagulation implemented after the BVOS [2], we found heterogeneous control groups ranging from sham injection in BRAVO to grid laser photocoagulation in SCORE-BRVO [4,23]. BRAVO allowed grid as rescue therapy, which complicates the indirect comparison of the trials. Only one trial compared grid laser photocoagulation in combination to intravitreal bevacizumab 1.25 mg to bevacizumab alone, and found even better results in the combination group (+20 letters [median 3 injections]) [29]. There are currently 4 trials registered that compare intravitreal ranibizumab with macular laser photocoagulation and/ or a combination therapy (RABAMES, NCT00562406; BRIGHTER, NCT01599650; NCT01189526, clinicaltrials.gov, and EUCTR2008-007175-24-HU, www.who.int/ictrp/en/), and 1 RCT comparing triamcinolone versus grid versus combination (ChiCTR-TRC-05000661, www.who.int/ictrp/en/). A major limitation of all studies with grid laser photocoagulation is the heterogeneity of the treatment itself and the lack of evaluation of grid by the reading centers. 

#### Two year data

Two year data are only published for ranibizumab within the course of the HORIZON trial [30]. But interpretation of data is limited by the high drop-out rate in all groups and the early termination after approval for ranibizumab. Patients received significantly less intravitreal injections during the second year (CRVO 3.5 injections, BRVO 2.1) and lost little visual acuity BCVA (CRVO -4 letters, BRVO -0.7 letters) [30]. Two years follow-up of COPERNICUS [20] and GALILEO [33] will provide us with more evidence regarding anti-VEGF in CRVO.

#### Clinically significant gain in BCVA (more than 15 letters)

Analysing SCORE, COPERNICUS, CRUISE, BRAVO and Epstein studies, anti-VEGF treatment of macular edema leads to a clinically significant visual improvement in almost twice as many participants as triamcinolone or grid laser photocoagulation. Approximately 25% responders were found for triamcinolone [4,5], while anti-VEGF trials found 50% [22], 55% [20] and 60% [26] in CRVO (Figure 2) and 60% in BRVO [23] (Figure 3). Results at 12 months in control groups are better than after 6 months and the differences between drug and control groups smaller, because patients were treated open-label with the investigational product for months 6 to 12 in all multicenter trials (CRUISE, BRAVO, Epstein, COPERNICUS, GENEVA) [8,20,22,23,26]. Our analysis of 12 months data still shows a striking difference over all anti-VEGF trials between the groups, which were treated with the individual therapeutic agent from the beginning and the control/PRN groups, which had a delay of 6 months before therapy started. In conclusion, the effect of the anti-VEGF agents on macular edema in RVO is more pronounced if treatment starts early after onset of macular edema. 

#### Time between symptoms and treatment

On basis of these results, one can assume that time between occlusion and treatment is a critical factor both for patient choice in the context of clinical trials but also with regard to the therapeutic effect and the interpretation of study data. In practice, studies including and treating patients shortly after RVO onset might find better visual acuity outcome and/ or response to treatment than trials including old RVO with persistent macular edema, resulting in false negatively reduced outcome. Therefore, we specifically depicted time of occlusion in the list of study characteristics. Both, mean time from occlusion and percentage of patients with ≤ 2-3 months occlusion time differed between the included RCTs. One explanation is the choice of time span for inclusion criteria. In CRVO trials, mean time of occlusion ranged from 1.9 months (bevacizumab, Epstein 2012), 1.9 and 2.7 (aflibercept, COPERNICUS), 3.3 (ranibizumab, CRUISE), to 4.2 (triamcinolone, SCORE) and 5.2 months (dexamethasone implant, GENEVA) (Table 1) [4,5,8,20,22,26]. We found that the time to treatment is considerably longer in the trials investigating steroids compared to anti-VEGF trials. This is also expressed in high percentages of “fresh” RVO ≤ 2 months of 56-71% in COPERNICUS, RVO ≤ 3 months of 69% in CRUISE and only 15-18% in GENEVA (Table 1) [8,20,22]. The same discrepancy was found in BRVO trials, the percentage of “fresh” RVO was 65% in BRAVO and 37% in SCORE [23,34]. This might put into perspective the differences in visual acuity outcome between the RCTs. 

#### Ocular adverse events

The rate of endophthalmitis was very low in all RCTs (<1%) regardless of the substance class. SCORE-BRAVO, GENEVA, Epstein, and CRUISE did not find any endophthalmitis in approximately 6000 intravitreal injections (Table 3) [4,8,22,26]. These findings in RVO are in accordance to safety results and analysis of ranibizumab and bevacizumab in AMD [35]. Due to the different frequency of injection between anti-VEGF (8 injections/ 12 months) and steroids (2 injections/ 12 months) the risk of endophthalmitis for the individual patient is higher in an anti-VEGF treatment regimen than in triamcinolone or dexamethasone. 

IOP increase and medical treatment of IOP occurred more frequently in SCORE and GENEVA than in any anti-VEGF trial (Table 4). Looking into SCORE data, IOP increase seems to be dose-dependent with maximum rates of 35% (CRVO) and 41% (BRVO) for triamcinolone 4 mg [4,5]. GENEVA found approximately 25% in all dexamethasone implants after 6 months. It remains unclear, whether the 10% rate reported for 6-12 months data are in addition to these 25% [8,9]. No need for medical or surgical IOP control was reported in Epstein, CRUISE, BRAVO and HORIZON (Table 4) [22,23,26,30]. COPERNICUS found IOP increase in all groups of > 10%, which needed no medical treatment [20,36]. Rates of glaucoma surgery was occasional in GENEVA (1.3%, all dexamethasone implants), low in SCORE (2.2% CRVO triamcinolone 4 mg) and COPERNICUS (2.7% sham group, 1.7% sham/aflibercept 2 mg), and not reported for bevacizumab or ranibizumab trials [5,8,20,22,23,26]. It is remarkable, that in SCORE and GENEVA trials only participants with treatment of steroids developed glaucoma which needed surgery, while in anti-VEGF trials participants did not need surgery under ranibizumab or bevacizumab treatment [4,5,8,22,23,26]. Additionally, in COPERNICUS, surgery had to be performed in the sham/aflibercept group, probably due to secondary neovascular glaucoma [20]. 

Rates of cataract and surgery were found more frequently in SCORE and GENEVA with 20 - 35% cataract progression, compared to none in anti-VEGF trials (Table 4) [4,5,8]. 

Vitreous hemorrhages, either due to the injection procedure or because of progression to neovascular RVO, was found seldom in all treatment groups. We found a tendency towards higher rates in control groups in CRVO, which received treatment 6 months later. This difference was most pronounced in COPERNICUS (0.9% [aflibercept] versus 7.1% [sham/aflibercept]), and less striking in CRUISE (5.3% [ranibizumab 0.3/0.5 mg], 5.4% [0.5/0.5 mg] versus 8.8% [sham/0.5 mg]) [20,22]. GENEVA did not report vitreous hemorrhages [8,9]. In BRVO no difference between treatment and control groups was found [23,30]. This finding may be explained by the higher risk of CRVO for vitreous hemorrhage compared to BRVO due to the bigger area of tissue affected [1,2]. 

In conclusion, physicians have to take these differences in ocular risk profiles between anti-VEGF and steroids into account to choose the best therapy for their individual patients. 

#### Systemic adverse events

The systemic risk seems to be comparable low between all compounds given intravitreally in RVO. Rates of death were low (0-3%) over all groups and no statistically significant difference was found (Table 5). Comparison of systemic adverse event rates was biased by different reporting and difficulty in data extraction. None of the RCTs investigated in this review found significant differences within cerebrovascular events, non-ocular hemorrhages or infections. Infections were found in up to 7.9% in anti-VEGF trials (all groups, all substances), but more often in SCORE patients (10-19% all groups) [4,5,20,30]. Rates of infections are higher than reported before in ranibizumab (2-3.8%) or bevacizumab (4-6%) [14,35]. One has to take into account that RVO patients tend to be younger than AMD patients, but present more often with cardiovascular diseases [37]. Hypertension, diabetes, hypercholesterolemia and adiposity contribute to RVO but not to AMD [37,38]. Thus it is almost surprising that RVO trials do not find more myocardial infarction or cerebrovascular events than AMD trials. A possible explanation might be age as risk factor for both. Another explanation is the selection bias at time of inclusion within a RCT. Individuals presenting with considerable systemic diseases in their recent past medical history are most often excluded. Certainly, in all trials numbers of participants are still too low to be calculated to detect small differences in rare (systemic) events. 

#### Strengths and Limitations

We limited our search to RCTs and outcome data of at least 12 months to strengthen the implication for clinical practice as RVO treatment seeks an ongoing intervention. To investigate efficacy and safety outcome on best available data, we just referred to RCTs. We anticipated a vast number of reports of off-label bevacizumab use in RVO similar to the publication mode of AMD trials. Regarding safety data, previous analysis of similar reports often found a lack of monitoring and reporting of adverse events [35]. These shortcomings were also found in our review. Overall, we found better reporting and analysis in multicenter RCTs than in monocenter trials. 

Our search detected no head-to-head studies comparing two different anti-VEGF agents or anti-VEGF vs. steroids. This is a major limitation when comparing the therapeutic outcome and prevents us from taking clear recommendation for clinical choice. Only direct comparison within the same trial leads to sufficient evidence due to the same criteria for all participants. Regarding RVO, comparison of baseline characteristics is crucial due to the pathophysiology and natural course of macular edema in RVO. In our opinion, time between occlusion and start of treatment is a major confounder comparing published trials. As discussed above, the differences in rates of “young” versus “old” RVO between anti-VEGF and steroids treated patients may contribute to the differences seen in outcome. Data extraction was biased by different or no reporting of important outcomes, like the percentage of patients with gain or loss of 15 letters at 12 months. This is partly due to the different choice of primary end points between the trials. Complicating data retrieval, some data on CRVO and BRVO were published combined, e.g. outcome of dexamethasone treatment [8]. These limitations can only be overcome by trials comparing 2 or more substances directly. Fortunately, several head-to-head trials started recently to investigate dexamethasone implant versus bevacizumab (NCT01231633, www.who.int/ictrp/en/) or ranibizumab (COMRADE-B/ -C/ Extension, NCT01396057, NCT01396083, NCT01580020, and COMO, NCT01427751, www.who.int/ictrp/en/). Notably, COMRADE trials investigate BRVO and CRVO separately, and COMO is a head-to-head trial for BRVO. Additionally, we found two trials addressing the question of costs and effectiveness comparing bevacizumab vs. ranibizumab in RVO (NCT01428388, NCT01635803, www.who.int/ictrp/en/).

On the other hand, we see the strengths of our systematic review in the critical and detailed data extraction of all 12 months data on anti-VEGF and steroids available in the current literature. Analysis of the detected RCTs shows both remarkably high validity of efficacy and safety for many of these trials which strengthens the quality of reported outcomes and supports comparison of outcomes. To our knowledge this review is the first to report and compare 12 months safety and efficacy data of five therapeutic agents currently in use for macular edema in RVO. This is of great value to all ophthalmologists to help decide between the therapeutic options and to calculate the accompanying risks.

#### Other reviews

We identified only 1 Cochrane review on anti-VEGF for macular edema secondary to BRVO [39] and 1 review evaluating CRVO [40]. Both reviews limited their search and outcome reporting on anti-VEGF agents without considering steroids. In addition, their results are based on data of only 2 (quasi-)RCTs. In contrast, our systematic review includes 11 up-to-date RCTs investigating and comparing different therapeutic (anti-VEGF and steroids) options with follow-ups of at least 12 months. Therefore, the current systematic review functions as a detailed update on knowledge of treatment for macular edema in both, BRVO and CRVO. 

#### Implications for Clinical Practice

Our search detected 5 trials in CRVO, each of them providing evidence for the superiority of triamcinolone, dexamethasone implant, bevacizumab, ranibizumab, and aflibercept, respectively [5,8,20,22,26], in comparison to observation or sham, which was the unsatisfying gold standard before initiating intravitreal therapy [1]. In BRVO, ranibizumab has been established as the new gold standard, even if it received FDA approval without testing it against the grid laser photocoagulation (the previous gold standard for this indication) [2,23]. Smaller trials implicate superiority of bevacizumab [25,26] or ranibizumab (RABAMES, www.clinicaltrials.gov) against grid [28,29]. But more evidence is needed to judge, whether patients may profit from a combination or change in therapeutic strategy. 

Regarding head-to-head studies, we look forward to ongoing clinical trials such as COMO and COMRADE, comparing ranibizumab directly with dexamethasone implant (www.clinicaltrials.gov). We also expect vast use of off-label bevacizumab due to monetary necessities as seen in AMD and appreciate scientific evidence such as given by Epstein for bevacizumab in CRVO [26,35,41,42]. Given the different medical background of RVO compared to AMD patients, we should not uncritically transfer results from comparative CATT and IVAN trials on the question of off-label use of bevacizumab versus ranibizumab in RVO [14,15]. RVO patients may present with a different spectrum of underlying diseases and potentially higher risk profiles. Evidence from head-to-head studies addressing the question of costs and effectiveness comparing bevacizumab versus ranibizumab in RVO (NCT01428388, NCT01635803, www.who.int/ictrp/en/) is needed to judge upon safety and efficacy in RVO. 

In conclusion, macular edema in CRVO and BRVO responds to intravitreal therapy of steroids and various anti-VEGF agents. Best visual acuity results at 1 year are found after aflibercept 2 mg and bevacizumab 1.25 mg in CRVO, and ranibizumab 0.5 mg in BRVO. Cataract and glaucoma are the main draw-backs in the use of triamcinolone and dexamethasone, while low injection frequency is in favor of steroids. To our knowledge, this review is the first to report 12 months data of five different therapeutic intravitreal agents currently used in RVO. Therefore, these findings may be of great value for all ophthalmologists.

## Supporting Information

Text S1 **Search strategy in Medline (Ovid).**
(RTF)

Table S1 **Preferred Reporting Items for Systematic Reviews and Meta-Analyses (PRISMA) checklist.**
(DOC)
