# Supplementary material for: Efficacy and Safety of Intravitreal Therapy in Macular Edema Due to Branch and Central Retinal Vein Occlusion: a Systematic Review
Source: PLoS One. 2013 Oct 25;8(10):e78538. doi: 10.1371/journal.pone.0078538 (PMC3808377; doi:10.1371/journal.pone.0078538)
Supplement: Text S1 — Search strategy in Medline (Ovid). (RTF) [file pone.0078538.s001.rtf]

Search strategy in Medline und Premedline (Ovid) on April 12th 2011
  
1. exp Retinal Vein Occlusion/
2. exp Retinal Vein/
3. retinal vein occlusion.mp.
4. retinal vein.mp.
5. ((vein* or occlu* or obstruct* or clos* or stricture* or steno* or block* or embolism*) adj3 retina*).mp. [mp=protocol supplementary concept, rare disease supplementary concept, title, original title, abstract, name of substance word, subject heading word, unique identifier]
6. branch retinal vein occlusion.mp.
7. central retinal vein occlusion.mp.
8. central vein occlusion.mp.
9. (BRVO or CRVO or RVO).mp. [mp=protocol supplementary concept, rare disease supplementary concept, title, original title, abstract, name of substance word, subject heading word, unique identifier]
10. 1 or 2 or 3 or 4 or 5 or 6 or 7 or 8 or 9
11. exp Steroids/
12. steroid*.mp.
13. exp Glucocorticoids/
14. glucocorticoid*.mp.
15. exp Triamcinolone Acetonide/ or exp Triamcinolone/
16. Triamcinolone.mp.
17. exp Dexamethasone Isonicotinate/ or exp Dexamethasone/
18. dexamethasone.mp.
19. ozurdex.mp.
20. dex implant.mp.
21. 11 or 12 or 13 or 14 or 15 or 16 or 17 or 18 or 19 or 20
22. exp Antibodies, Monoclonal/
23. monoclonal antibod*.mp.
24. exp Angiogenesis Inhibitors/
25. Angiogenesis inhibit*.mp.
26. exp Angiogenesis Inducing Agents/
27. angiogenesis inducing agent*.mp.
28. exp Endothelial Growth Factors/
29. ((endothelial adj3 growth factor*) or endo-GF).mp. [mp=protocol supplementary concept, rare disease supplementary concept, title, original title, abstract, name of substance word, subject heading word, unique identifier]
30. exp Vascular Endothelial Growth Factors/
31. vegf.mp.
32. (anti adj2 vegf*).mp. [mp=protocol supplementary concept, rare disease supplementary concept, title, original title, abstract, name of substance word, subject heading word, unique identifier]
33. bevacizumab.mp.
34. avastin.mp.
35. ranibizumab.mp.
36. rhufab.mp.
37. lucentis.mp.
38. pegaptanib.mp.
39. macugen.mp.
40. 22 or 23 or 24 or 25 or 26 or 27 or 28 or 29 or 30 or 31 or 32 or 33 or 34 or 35 or 36 or 37 or 38 or 39
41. 10 and 21
42. 10 and 40
43. 41 or 42
44. 11 or 12 or 13 or 14 or 15 or 16 or 17 or 18 or 19 or 20 or 22 or 23 or 24 or 25 or 26 or 27 or 28 or 29 or 30 or 31 or 32 or 33 or 34 or 35 or 36 or 37 or 38 or 39
45. 10 and 44
46. Animals/
47. Humans/
48. 46 not (46 and 47)
49. 45 not 48
50. remove duplicates from 49
